# Comparative genomics reveals extensive intra-species genetic divergence of the prevalent gut commensal *Ruminococcus gnavus*

**DOI:** 10.1099/mgen.0.001071

**Published:** 2023-07-24

**Authors:** Rashidin Abdugheni, Chang Liu, Feng-Lan Liu, Nan Zhou, Cheng-Ying Jiang, Yonghong Liu, Li Li, Wen-Jun Li, Shuang-Jiang Liu

**Affiliations:** ^1^​ State Key Laboratory of Desert and Oasis Ecology, Key Laboratory of Ecological Safety and Sustainable Development in Arid Lands, Xinjiang Institute of Ecology and Geography, Chinese Academy of Sciences, Urumqi 830011, PR China; ^2^​ State Key Laboratory of Microbial Technology, Shandong University, Qingdao 266000, PR China; ^3^​ State Key Laboratory of Microbial Resources and Environmental Microbiology Research Center (EMRC), Institute of Microbiology, Chinese Academy of Sciences, Beijing 100101, PR China; ^4^​ College of Life Sciences, Hebei University, Baoding 071000, PR China; ^5^​ University of the Chinese Academy of Sciences, Beijing 100049, PR China; ^6^​ State Key Laboratory of Biocontrol, Guangdong Provincial Key Laboratory of Plant Resources and Southern Marine Science and Engineering Guangdong Laboratory (Zhuhai), School of Life Sciences, Sun Yat-Sen University, Guangzhou 510275, PR China

**Keywords:** antimicrobial-resistance genes, comparative genomics, phylogenomics, *Ruminococcus gnavus*, virulence factors

## Abstract

*
Ruminococcus gnavus* is prevalent in the intestines of humans and animals, and ambiguities have been reported regarding its relations with the development of diseases and host well-being. We postulate the ambiguities of its function in different cases may be attributed to strain-level variability of genomic features of *R. gnavus *. We performed comparative genomic and pathogenicity prediction analysis on 152 filtered high-quality genomes, including 4 genomes of strains isolated from healthy adults in this study. The mean G+C content of genomes of *R. gnavus* was 42.73±0.33 mol%, and the mean genome size was 3.46±0.34 Mbp. Genome-wide evolutionary analysis revealed *R. gnavus* genomes were divided into three major phylogenetic clusters. Pan–core genome analysis revealed that there was a total of 28 072 predicted genes, and the core genes, soft-core genes, shell genes and cloud genes accounted for 3.74 % (1051/28 072), 1.75 % (491/28 072), 9.88 % (2774/28 072) and 84.63 % (23 756/28 072) of the total genes, respectively. The small proportion of core genes reflected the wide divergence among *R. gnavus* strains. We found certain coding sequences with determined health benefits (such as vitamin production and arsenic detoxification), whilst some had an implication of health adversity (such as sulfide dehydrogenase subunits). The functions of the majority of core genes were unknown. The most widespread genes functioning in antibiotic resistance and virulence are *tetO* (tetracycline-resistance gene, present in 75 strains) and *cps4J* (capsular polysaccharide biosynthesis protein Cps4J encoding gene, detected in 3 genomes), respectively. Our results revealed genomic divergence and the existence of certain safety-relevant factors of *R. gnavus *. This study provides new insights for understanding the genomic features and health relevance of *R. gnavus *, and raises concerns regarding predicted prevalent pathogenicity and antibiotic resistance among most of the strains.

## Data Summary

The detailed information for the 157 public genome assemblies obtained from the GenBank database are presented in Table S1 (available with the online version of this article).

**Impact Statement**

*
Ruminococcus gnavus* is an important gut commensal, and its relevance in health revealed ambiguities. We analysed 152 filtered *R. gnavus* genomes, including 4 genomes we sequenced, and conducted a comparative genomic analysis regarding the pan–core genomes, phylogeny and the distribution of antibiotic-resistance and virulence genes. The results showed the small proportion of core genes, which reflects the wide divergence among *R. gnavus* strains. The coding sequences with health benefits (such as vitamin production and arsenic detoxification) and some with an implication of health adversity (such as sulfide dehydrogenase subunits) were also determined, but the functions of the majority of core genes were unknown. The tetracycline-resistance gene (*tetO*) and virulence gene *cps4J* were found in 75 and 3 genomes, respectively. This study revealed, for what is believed to be the first time, the biodiversity of the prevalent gut commensal *R. gnavus* from the genomic perspective, and presented the phylogenomic divergence and the distribution of antibiotic-resistant genes and virulence genes among the genomes of *R. gnavus *, all of which together improve our knowledge on *R. gnavus* from the genomic perspective.

## Introduction

*Ruminococcus gnavus* is a Gram-positive, strictly anaerobic, mucin-degrading, gut bacterium, and is prevalent in the intestine of humans and animals with a relatively high abundance. Being one of the highly abundant groups commonly found in the intestinal tracts of humans and animals [1–4], *R. gnavus* was naturally considered a harmless or neutral gut commensal for its prevalence in hosts of different age groups [5, 6] and for its production of well-documented beneficial metabolites, including butyrate and lactate [7, 8]. Reports showed that *R. gnavus* strains have positive functions involving food digestion, including starch, maltose and fucosylated glycans metabolism [8–11], and antitumor effects against colorectal cancer [12]. An animal-model-based study found that *R. gnavus* mediated hepatic FGF21 expression underlying the anti-obesity effects of BupE on mice [13], and ameliorates atopic dermatitis by enhancing the Treg cells [14].

Despite its important positive side, in recent years, the adverse effects of *R. gnavus* on host health have been explored as well. *R. gnavus* has been reported to cause septic arthritis [15], bacteraemia [16] and bloodstream infection [17]. High-throughput sequencing based studies found the association of *R. gnavus* with the occurrence of certain intestinal and other major diseases. Association of *R. gnavus* with adiposity [18], development of inflammatory bowel disease (IBD) [19] and Crohn’s disease [20], and moyamoya disease and non-moyamoya intracranial large artery disease [21], have been reported as well; hence, this important gut inhabitant may play a pathogenic role in diarrhoea-predominant irritable bowel syndrome [22]. Contradictory to previous reports, the relative abundance of *R. gnavus* in overweight/obese patients was found to be significantly increased [23].

Some of those previous reports were ambiguous and even controversial. Based on that, we postulate that the deleterious, beneficial or neutral effects of *R. gnavus* might be attributed to differences and divergence at the strain level, and may be explored and elucidated by genome-based investigations. However, until now, most of the studies focused on the relevance of *R. gnavus* with any cases or studies involving just a limited number of strains of *R. gnavus *, and there was no genome-based investigation considering the complete species *R. gnavus *. The exclusive relations, functions and mechanism behind these observations and findings remained to be explored.

To determine the genomic lineage, biodiversity and major features of the genomes of *R. gnavus *, we investigated and evaluated the phylogeny, genetic relatedness and genomic features of strains of *R. gnavus* using 161 genomes, which included 4 genomes we sequenced using four *R. gnavus* strains isolated from healthy individuals in the current study. We performed analysis on 152 filtered high-quality genomes, including feature determination, comparative genomics regarding the pan-genome and core-genome of *R. gnavus *, and prediction of virulence and antibiotic genes on these 152 genomes. The determined genomic lineage and divergence of *R. gnavus* in the current study provide an important understanding of the organism's variance from the genomic perspective, and raise concerns about its safety regarding the distribution of virulence and antibiotic genes, requiring further consideration of preventive or therapeutic measures for the potential clinical issues these may cause.

## Methods

### Isolation of four *R *. *gnavus* strains and genome sequencing

Four *R. gnavus* strains, namely RSHDN_120, RSHDN_121, RSHDN_122 and RSHDN_123, were isolated from the faeces of four healthy Chinese adults, during a massive cultivation of human gut microbes. Fresh faecal samples were collected and immediately transferred into an anaerobic workstation (AW 500SG; Electrotek) filled with 85 % N_2_, 5 % CO_2_ and 10 % H_2_, and isolation, culturing, purification, identification and preservation of micro-organisms were conducted according to our previous methods [24]. Genomic DNA was extracted from overnight bacterial cultures using a Wizard genomic DNA purification kit (Promega), following the manufacturer’s protocol, and assembled according to our previous report [25]. Genomes of the four strains RSHDN_120, RSHDN_121, RSHDN_122 and RSHDN_123 were submitted to the publicly available GenBank database, and genome features were determined using CheckM (version 1.1.3) [26].

### Genome quality assessment and calculation of the average nucleic acid identity (ANI)

**Fig. 1.**
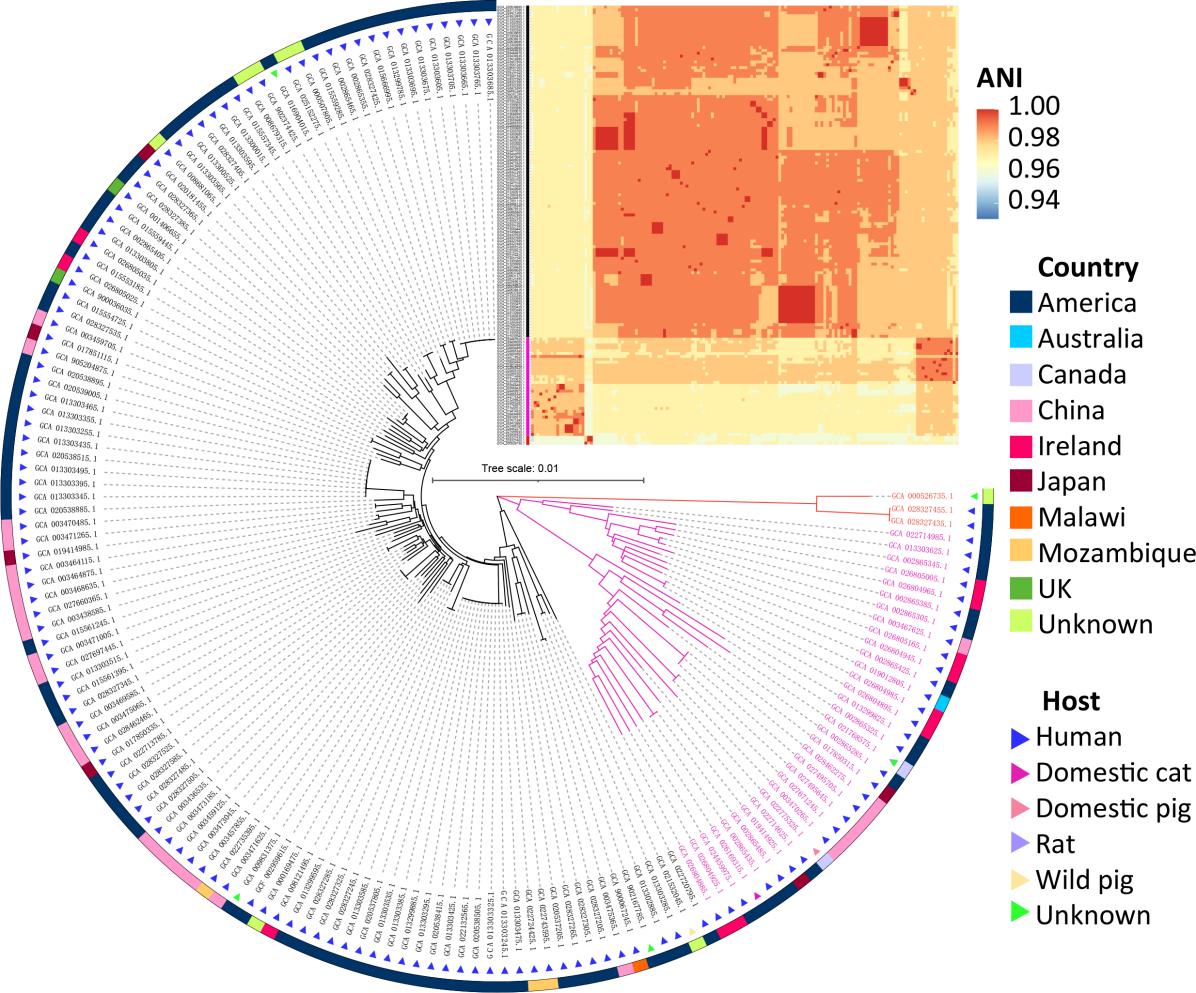
The core-gene-based phylogenetic tree and the whole-genome-based all-to-all ANI similarity assessment of the 152 *R *. *gnavus* genomes. Information about the genome source and the all-to-all ANI similarity index is presented. Bar, 0.01 substitutions per nucleotide position.

In addition to the sequenced genomes of four strains isolated in the current study, the publicly available genomes of *R. gnavus* (as of 30th January 2023) were downloaded from the National Center for Biotechnology Information (NCBI) [27] (Table S1), under the ‘Assembly’ section, querying for ‘* Ruminococcus gnavus *’, and the yielded accessions were stored in a ‘Genome.txt’ file. The genome assemblies were downloaded using the ncbi-genome-download tool (https://github.com/kblin/ncbi-genome-download/), by using the following script: ‘ncbi-genome-download --assembly-accessions Genome.txt bacteria --section genbank --formats fasta --flat-output’, and unzipped to obtain genome assemblies. The quality of the genomes regarding the completeness and contamination was assessed using CheckM (version 1.1.3) [26], and genomes with completeness above 90 % and contamination below 5 % were preliminarily considered as high-quality genomes. In addition, to further confirm the genome quality regarding the species criteria, the preliminarily filtered genomes were assessed using an all-to-all ANI analysis using a Python script, pyANI (https://github.com/widdowquinn/pyani) [28], and genomes where the pairwise ANI values were above the species threshold of 95 % [29] were selected and used in further genomic analysis. An all-to-all ANI heatmap was generated using the Pheatmap package [30] by hierarchical clustering of both columns and rows with the ANI value matrix of the 152 filtered genomes, but the hierarchical trees were not presented, and the group labels were added for the three different lineages based on the ANI clustering (Fig. 1). The main features (i.e. genome size, G+C content and completeness, etc.) of the finally determined high-quality *R. gnavus* genomes were visualized using the ggplot2 package [31].

### Genome annotation, phylogenomics and pan−core genome analysis

The overall screened high-quality assemblies were annotated using Prokaryotic Genome Annotation System (Prokka) (version 1.12) [32], and the autoProkka script (https://github.com/stevenjdunn/autoprokka) using the default parameters. In order to facilitate in-depth evolutionary and population genetics analyses of these genomes, the ‘.gff’ files obtained from the Prokka annotation were used in pan-genome analyses by Roary (version 3.7.0) [33], and according to the presence and absence of the genes in the included strain genomes, core genes (99 %≤ strains ≤100 %), soft-core genes (95 %≤ strains <99 %), shell genes (15 %≤ strains <95 %) and cloud genes (0 %≤ strains <15 %) were determined. Results were visualized using the ggplot2 package [31] and the Pheatmap package [30]. The phylogenetic tree was edited using the Interactive Tree Of Life (iTOL, version 4.0, https://itol.embl.de) [34].

### Determination of virulence genes, antibiotic-resistance genes and plasmids

Genomic islands with antimicrobial-resistance (AMR) genes were predicted using ABRicate (version 0.8.7) (https://github.com/tseemann/abricate/) searches by the default parameters referring to the Antibiotic Resistance Gene-Annotation (ARG-ANNOT) database (version: July 8 2017) [35], the NCBI β-lactamase database, the Comprehensive Antibiotic Resistance Database (CARD; https://card.mcmaster.ca/) (version: March 17 2017) and the ResFinder database (version: July 8 2017) [36]. In order to remove false positives, we applied a cut-off of the best identity of >75 % (as default) [37]; and to confirm the validity of the result, we applied >80 % length of the query coverage as in AMR++ (version 3.0) [38] to present as the final result of the determination, and results visualized accordingly. Virulence factors were screened referring to the Virulence Factor Database (VFDB) (version: March 17 2017) [37], and the plasmids were predicted referring to the PlasmidFinder database using ABRicate (version 0.8.7) as well*,* and results were visualized using the Pheatmap package [30].

## Results

### Genomes and genomic features of *R *. *gnavus*

The genomes of the strains RSHDN_120, RSHDN_121, RSHDN_122 and RSHDN_123 were submitted to GenBank under the accession numbers GCA_028462465.1, GCA_027495645.1, GCA_028462275.1 and GCA_027495705.1, respectively. The basic genome features are presented in Table 1.

**Table 1.** Genome features of the four strains recovered from human faeces

Genome feature	RSHDN_120	RSHDN_121	RSHDN_122	RSHDN_123
GenBank accession no.	GCA_028462465.1	GCA_027495645.1	GCA_028462275.1	GCA_027495705.1
Genome size (Mbp)	3.39	3.04	3.12	3.15
G+C content (mol%)	42.6	42.7	42.6	42.6
Completeness (%)	99.42	99.42	99.42	99.41
No. of contigs	63	65	67	65
N50 of contigs (bp)	160 135	198 019	198 117	198 117
No. of genes	3278	2889	2981	2983

Also, we obtained 157 additional genomes from the GenBank database, which included metagenomic assemblies (*n*=29), a single-cell extracted genome (*n*=1) and cell-culture extracted genomes (*n*=124). The basic information of the overall 161 genomes is detailed in Tables S1 and S2.

After the CheckM filtration, there primarily remained 153 high-quality genomes (Table S2). By all-to-all ANI calculation, we found a genome (GenBank accession no. GCA_020538135.1) that shared less than 75 % ANI identity (Table S3), which was below the proposed prokaryotic species delineation threshold of 95 % [29, 39]. We also conducted the digital DNA–DNA hybridization (dDDH) analysis of this genome against the genome of the type strain of *R. gnavus* (strain ATCC_29149^T^, GenBank accession no. GCA_002959615.1) using the online dDDH calculator (https://ggdc.dsmz.de/ggdc.php) [40], and the results yielded from the formula 2 was 22.10 %, which was below the species threshold of 70 % [29]. So the genome was eliminated, and not included in the further analysis. Finally, there remained a total of 152 confirmed high-quality genomes with which to conduct analyses. The 152 filtered genomes were isolated from the following: humans (*n*=144), domestic and wild pigs (*n*=1, *n*=1), rat (*n*=1), domestic-cat-related samples (*n*=1) and other unknown samples (*n*=4). These genomes were obtained from America (*n*=91), Australia (*n*=1), Canada (*n*=2), China (*n*=27), Ireland (*n*=11), Japan (*n*=6), Malawi (*n*=1), Mozambique (*n*=3) and the UK (*n*=2), and there were also some genomes (*n*=8) for which the host and origin information were not available.

The major features of the 152 genomes are presented in Fig. S1. The genome sizes varied from 2.56 to 4.20 Mbp across strains (a mean of 3.46±0.34 Mbp), and the average G+C content of genomes of *R. gnavus* varied from 41.22 to 43.89 mol% (with a mean of 42.73±0.33 mol%), which shows that *R. gnavus* genomes varied significantly in size, and G+C content and genome completeness, which primarily indicates the divergence of strains of *R. gnavus* at the genome level.

### Phylogenetics, pan-genome and core-genome of *R *. *gnavus*

A phylogenetic tree reconstructed from 1051 core genes by Roary showed that they were present in all 152 *R *. *gnavus* genomes, and it revealed that the 152 genomes clustered into three major clades, and several divergent clusters. The ANI value-based heatmap of the 152 *R *. *gnavus* genomes denoted the species-level identity and three major lineages of the genomes (Fig. 1), which indicated the genomic variance of *R. gnavus *.

**Fig. 2.**
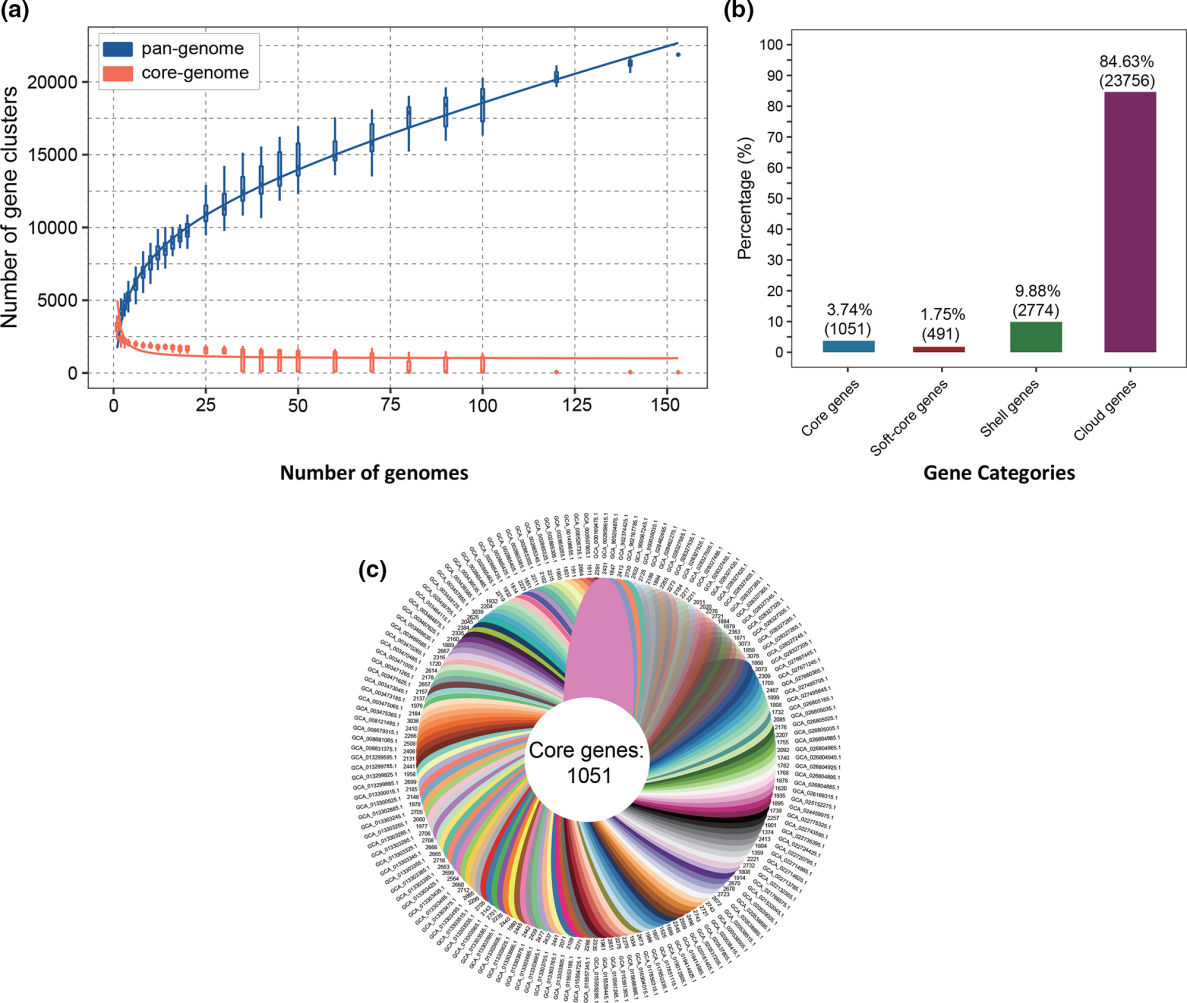
Pan-genome analyses of 152 *R *. *gnavus* genomes. (**a**) The pan-genome is represented by the accumulated number of new genes against the number of genomes added. The core-genome is represented by the accumulated number of genes attributed to the core-genomes against the number of added genomes. The red boxes denote the number of core genes discovered with the sequential addition of new genomes. (**b**) Gene occurrence plot of the core genes, soft-core genes, shell genes and cloud genes of *R. gnavus *. (**c**) Genomic diversity of 152 *R *. *gnavus* strains. Each strain is shown as a leaf. The number of core genes is shown in the centre. Strain-specific genes conserved only within a strain are presented at the ends of the leaves. The genome accession number of a strain is presented beside the leaf.

**Fig. 3.**
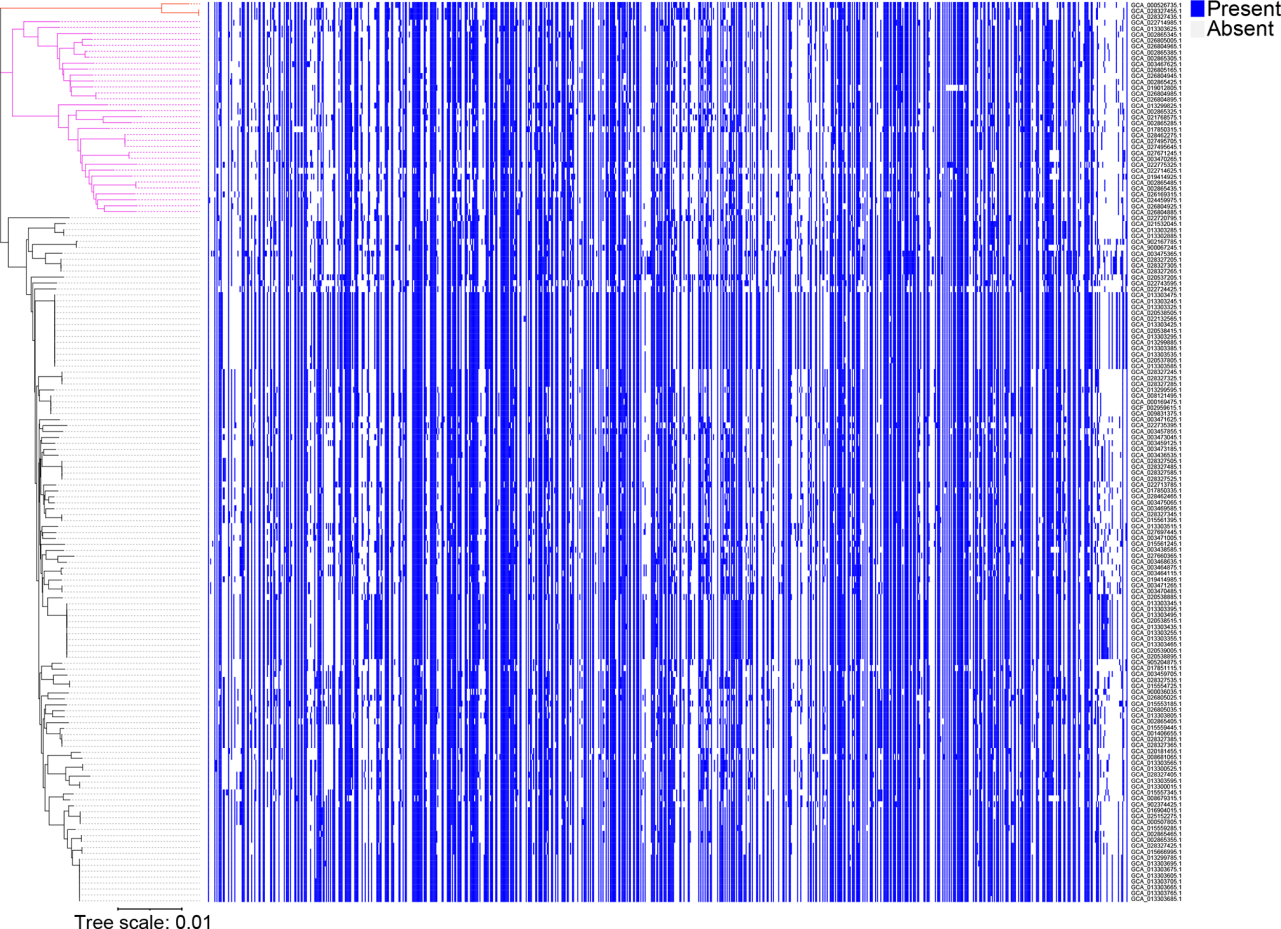
Pan-genome plot representing the gene presence/absence across the strains. Core-genome phylogroups are represented on the left as three clades coded in red, pink and black. In the plot, blue represents the presence of the gene(s), while light grey represents the absence of the gene(s) according to the results of the pan-genome analysis. Bar, 0.01 substitutions per nucleotide position.

Pan-genome analysis was performed to determine the total number of different genes which were present in the *R. gnavus* genomes. The gene accumulation curve showed that the numbers of the core-genome genes decreased continually with the addition of new strains, while the pan-genome showed an increasing trend (Fig. 2a). We also used a pan-genome plot representing the gene presence/absence across the strains to show the genomic lineage of the strains (Fig. 3, Table S4). The change of both curves slowed down because the pan-genome of *R. gnavus* was in an open state, indicating that unique genes would be added along with the addition of new strains.

The gene occurrence plot showed that a core-genome containing 1051 genes was present in all 152 *R *. *gnavus* strains. Pan-genome analyses of 152 *R *. *gnavus* genomes conducted by Roary revealed that there was a total of 28 072 genes predicted, and the number of core genes, soft-core genes, shell genes and cloud genes were determined to be 1051, 491, 2774 and 23 756, respectively (Fig. 2b). The number of core genes and the number of strain-specific genes of each strain, which are defined as the non-overlapping genes among genomes, are indicated in the flower chart (Fig. 2c). Collectively, pan-genome analysis using Roary resulted in a comparatively small core-genome (1051 genes) and a large pan-genome (27 021 genes), which reveals a wide diversity of gene content within the species *R. gnavus *. Results of core-genome analysis showed a host beneficial side from the genomic perspective in the current study, where we found certain coding sequences with determined health benefits, such as vitamin production and arsenic detoxification (Table S5). In addition, we found a large portion of carbohydrate metabolism related genes, such as trehalose, maltose and rhamnose metabolism genes, in the core-genome of *R. gnavus* (Table S5), which was also stated as a beneficial side of this species in food digestion.

### Distribution of functional categories of core genes of *R *. *gnavus*

**Fig. 4.**
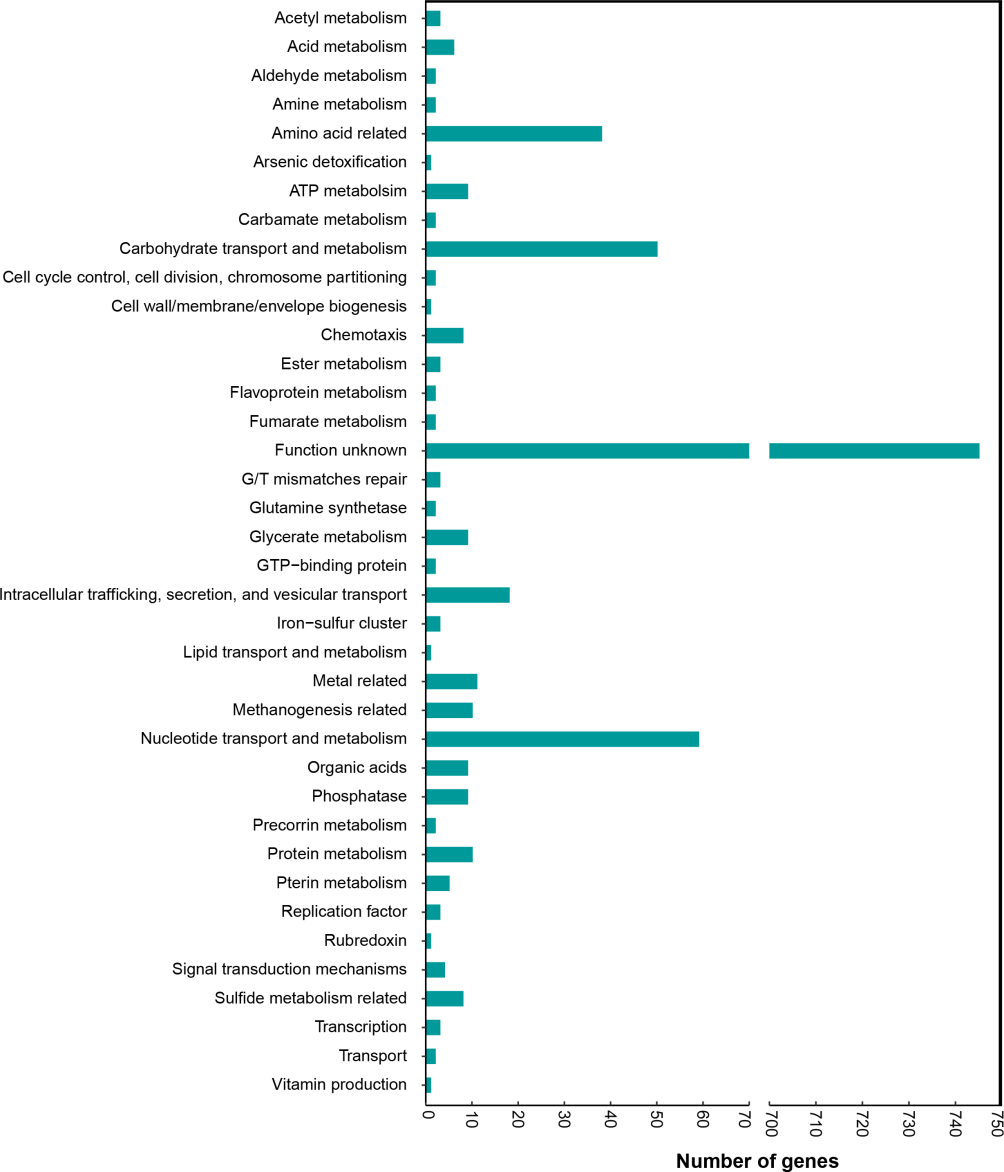
Distribution of functional categories of core genes of the 152 genomes of *R *. *gnavus *. The number of genes for the functional categories are presented on the *x*-axis.

According to the annotation results, we found that for the majority of the core genes function was yet-to-be-determined, but for the rest, the genes responsible for protein and amino acid metabolism, carbohydrate metabolism, and nucleic acid-related processes comprised the majority. We also found certain coding sequences with determined health benefits (such as vitamin production and arsenic detoxification) and some with an implication of health adversity (such as sulfide dehydrogenase subunits) (Fig. 4, Table S5). These results revealed that gene functions of *R. gnavus* remained to be explored, so as to determine its function in host health.

### Distribution of antibiotic-resistance and virulence-related genes

**Fig. 5.**
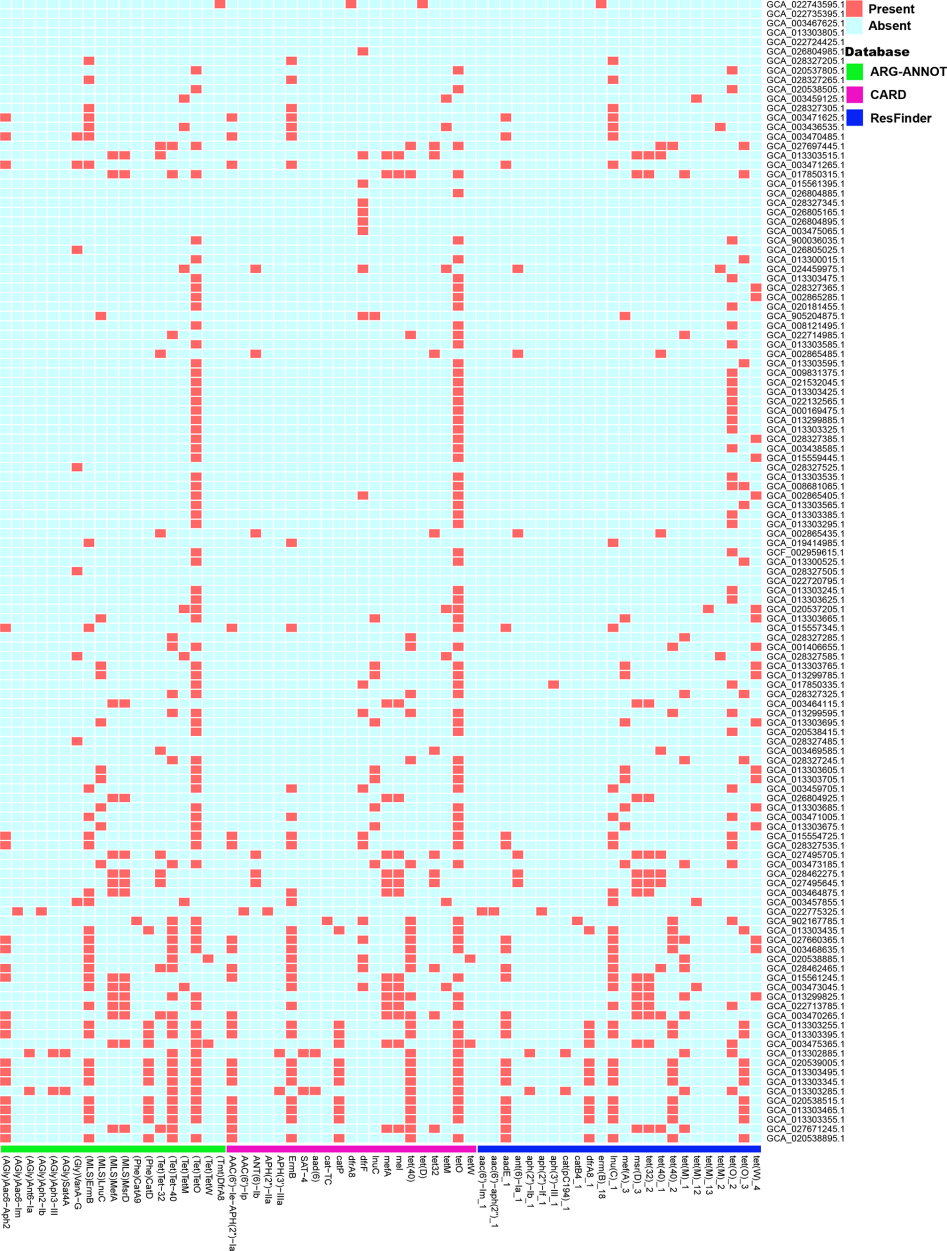
Determination of antimicrobial-resistance (AMR) genes in 152 *R *. *gnavus* genomes. Reference databases and presence/absence results are denoted as in the key. The red colour represents the presence of a drug-resistance gene.

A total of 73 antibiotic-resistance-related genes were identified in the 152 *R *. *gnavus* genomes using the ARG-ANNOT database, the NCBI β-lactamase database, the CARD database and the ResFinder database as references (Table S6). There are 31 genomes for which we did not detect any resistance or virulence genes (Table S6). Resistance genes for several general antibiotic classes were detected in over 30 genomes, including tetracycline-resistance-related genes (*tetO*, *tetW*, *tetM*, *msrD*) [41–43], macrolides, lincosamides, streptogramin B resistance-related genes (*lnuC*, *ermB*) [44, 45] and macrolide-resistance related genes (*mefA*) [46]. The overall results are presented in Fig. 5.

**Fig. 6.**
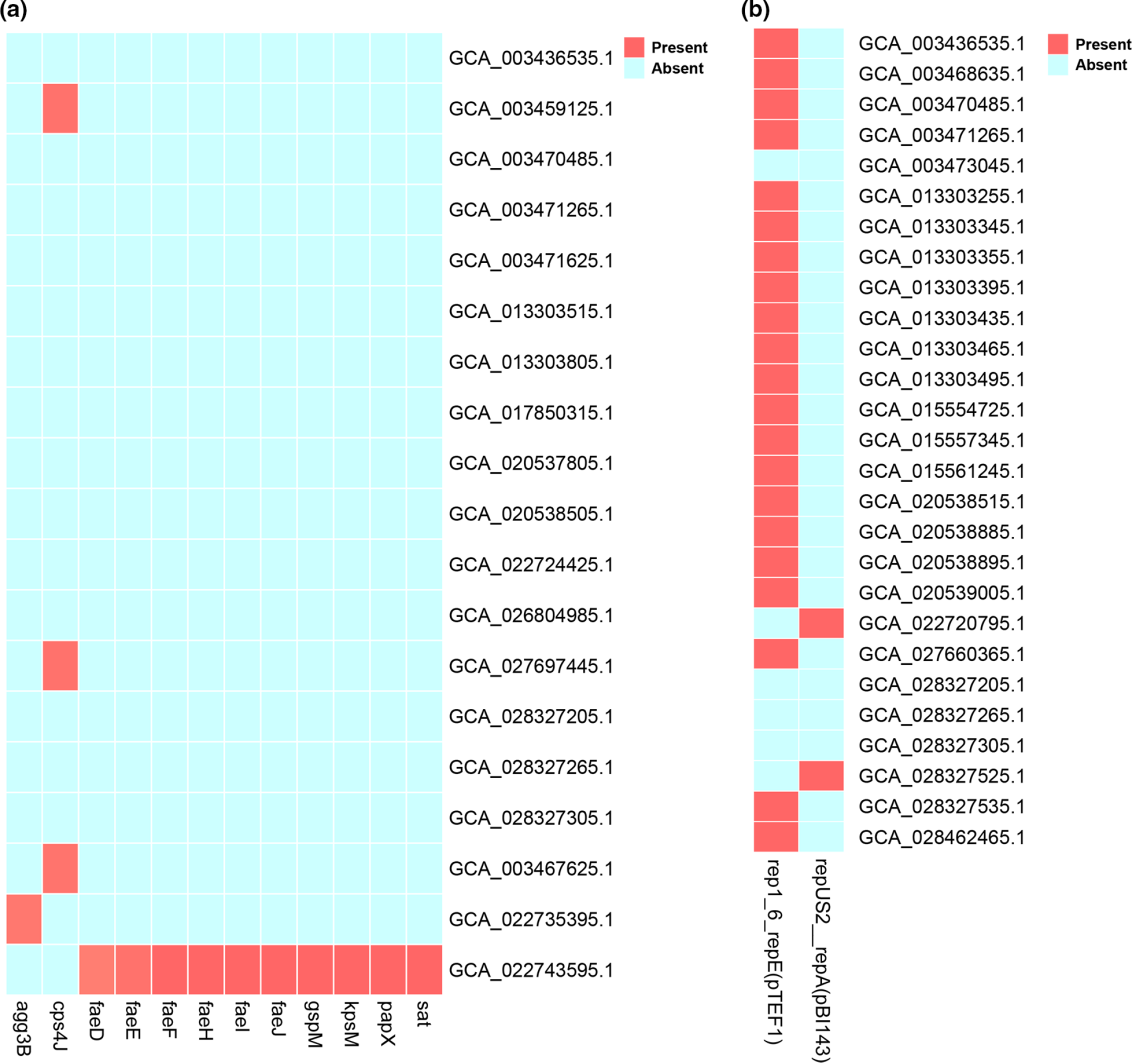
Distribution of the virulence factors and plasmids in 152 *R *. *gnavus* genomes. (**a**) Presence and absence of virulence factors. (**b**) Presence and absence of plasmids. Red and blue represents presence and absence of a gene, respectively.

We also detected several virulence-factor relevant genes, including secreted autotransporter toxin gene (*sat*), capsular polysaccharide encoding gene (*kpsM*), fimbrial protein-related genes (*faeJ*, *fael*, *faeH*, *faeF*, *faeE*, *faeD*), capsular polysaccharide biosynthesis protein Cps4J encoding gene (*cps4J*), which may be involved in the immunity regulation of *R. gnavus* [47], invasin protein-encoding gene (*agg3B*), and a gene encoding PapX protein that regulates flagellum synthesis to repress motility (*papX*) (http://www.mgc.ac.cn/VFs/search_VFs.htm) [48] (Fig. 6a). In addition, we detected plasmid pBI143, which included a tetracycline-resistance gene [49], and plasmid pTEF1, which may be relevant to vancomycin resistance [50] (Fig. 6b). Strains of *R. gnavus* were previously reported to be susceptible to penicillin, meropenem, tetracycline, metronidazole and clindamycin, and resistant to erythromycin, gentamicin, levofloxacin, moxifloxacin and tigecycline [51]. However, we detected tetracycline-resistance genes, and several virulence factors widespread in *R. gnavus *, which may explain the variance of beneficial and pathogenic sides of the species.

## Discussion

*R. gnavus* is one of the most abundant bacterial species in the intestinal tracts of humans and animals, and also an important inhabitant that has various significant but controversial impacts on host health. In this study, we included 152 high-quality genomes of *R. gnavus* after filtration of results, and the results of phylogenomic analysis showed the diversity of the *R. gnavus* members. The comparative genomic analysis showed that there was a total of 28 072 predicted genes, and the core genes, soft-core genes, shell genes and cloud genes accounted for 3.74 % (1051/28 072), 1.75 % (491/28 072), 9.88 % (2774/28 072) and 84.63 %(23 756/28 072) of the total genes. The small proportion of core genes reflected the wide divergence among *R. gnavus* strains, which was also stated in a previous study regarding the functional variety of the family *Lachnospiraceae* [52]. The significant difference in genomes implied the diversity of evolution and adaptation of *R. gnavus *, which may address its diverse impact on host health. *R. gnavus* adapts to different host intestinal environments by the advantage of utilization of carbohydrates [10], degradation of intestinal tissues and adhesion [53, 54], resistance to various antibiotics, and production of certain virulence factors, which contribute to the pathogenicity, persistence and advantage of *R. gnavus* in the long-term colonization of the gastrointestinal tract [55, 56].

*R. gnavus* showed a host beneficial side from the genomic perspective in that in the current study we found certain coding sequences with determined health benefits, such as vitamin production [57, 58] and arsenic detoxification [59]. In addition, we found some significant carbohydrate (trehalose and maltose) metabolism related genes in the core-genome of *R. gnavus *, which has also been stated as a beneficial side of this species in food digestion [60]. In addition, previous reports also stated the significant function of *R. gnavus* in bile acid metabolism, which is crucial to host health [61].

We also found genomic evidence of a detrimental side of *R. gnavus *. Our results showed that genes of sulfide dehydrogenase subunits exist in the *R. gnavus* pan-genome, and sulfide dehydrogenases were reported to be linked with health adversity. In addition, we found that the most widespread antibiotic-resistance and virulence genes among *R. gnavus* are *tetO* (tetracycline-resistance gene) and *cps4J* (capsular polysaccharide biosynthesis protein Cps4J encoding gene), respectively, which indicates the potential threat from these features. Nevertheless, not all the genomes of the strains contain a tetracycline-resistance gene, which indicates the tetracycline-resistance is not identical among *R. gnavus *, so the risky side of this species should be considered carefully. It’s notable that the functions of the majority of core genes are unknown, and remain to be mined in further studies. Detection of the enriched resistome profile of *R. gnavus* against important antibiotics may raise concerns about safety, and potential risks [62], and emphasize the value and importance of a genome-based approach to predict and evaluate the resistance development of gut commensals. Interestingly, as we also found rhamnose-related genes in the core-genome of *R. gnavus *, and generally, rhamnose is metabolized by anaerobes to produce host-beneficial chemicals such as butyrate [63, 64], but recent reports showed that *R. gnavus* produces an inflammatory polysaccharide, a complex glucorhamnan polysaccharide [20], by rhamnose metabolism, which raises concerns on the controversial roles of *R. gnavus* on host health.

The current work provides evidence for the genomic diversity and plasticity, and the divergent evolution, of *R. gnavus *, and is supported by the comparatively small number of core genes and the variety of genes for which the function is unknown. The results broaden our understanding of the evolution of this potential emerging human pathogen. In addition, this work will contribute to increasing the knowledge regarding the whole picture of this species from a genomic perspective.

## Supplementary Data

Supplementary material 1

Supplementary material 2

Supplementary material 3

Supplementary material 4

Supplementary material 5

Supplementary material 6

Supplementary material 7
