# Supplementary figures and images for: Comparative genomics reveals extensive intra-species genetic divergence of the prevalent gut commensal Ruminococcus gnavus
Source: Microb Genom. 2023 Jul 24;9(7):mgen001071. doi: 10.1099/mgen.0.001071 (PMC10438805; doi:10.1099/mgen.0.001071)

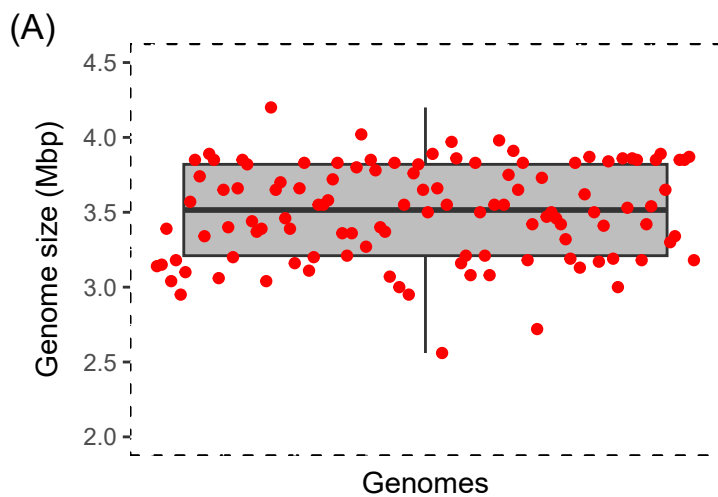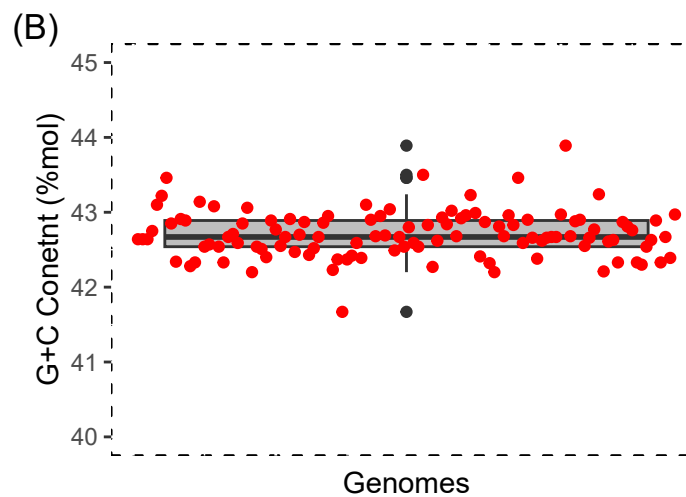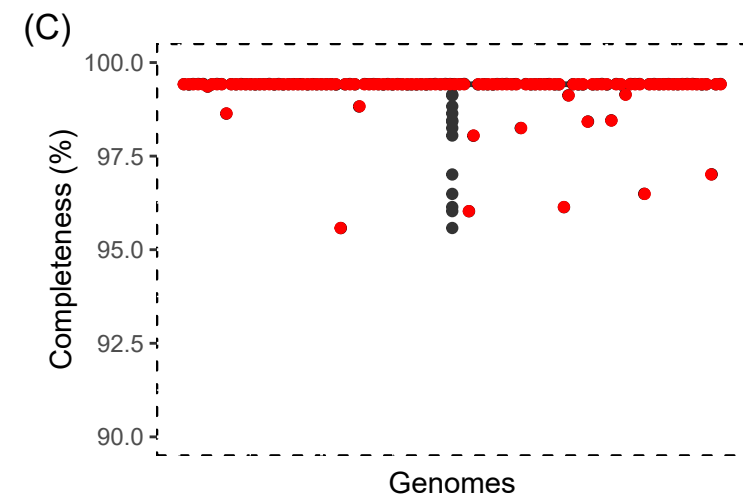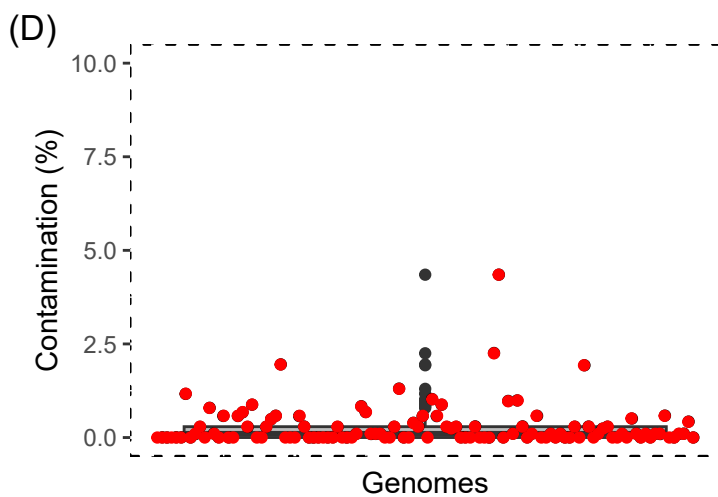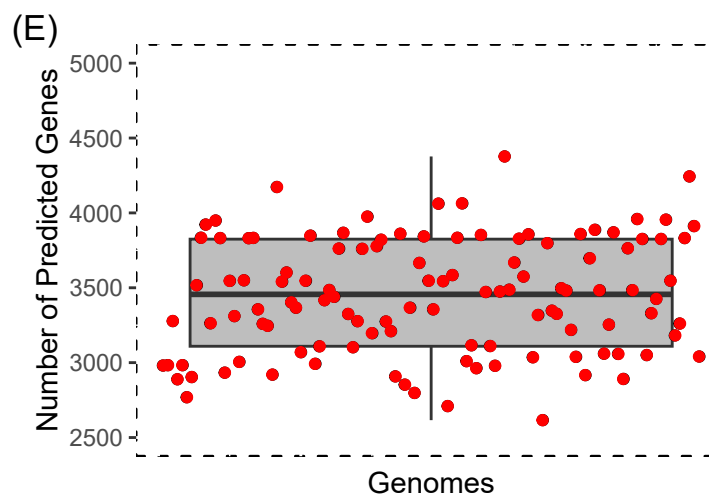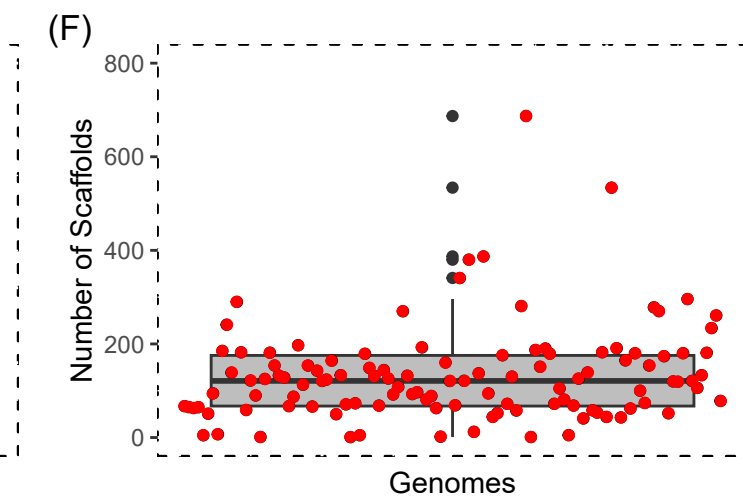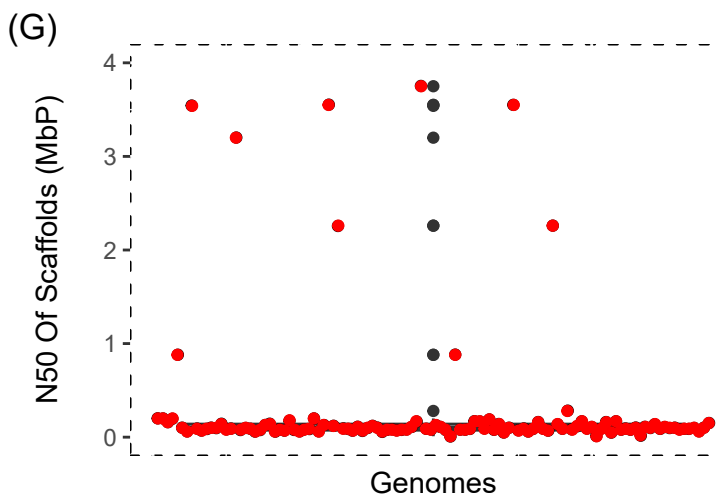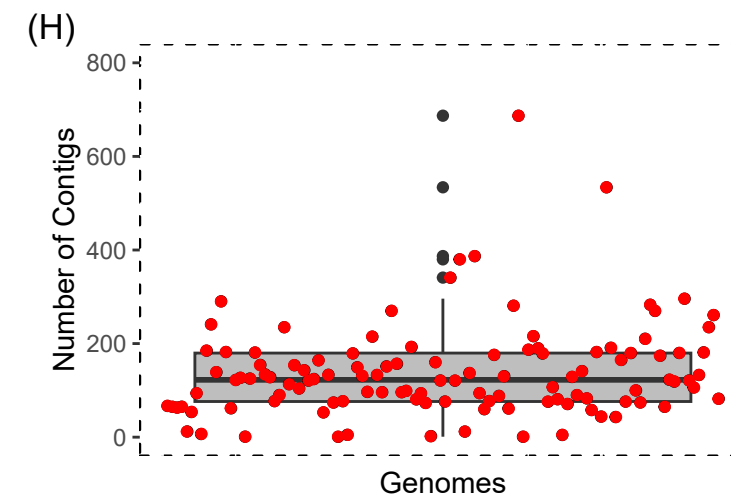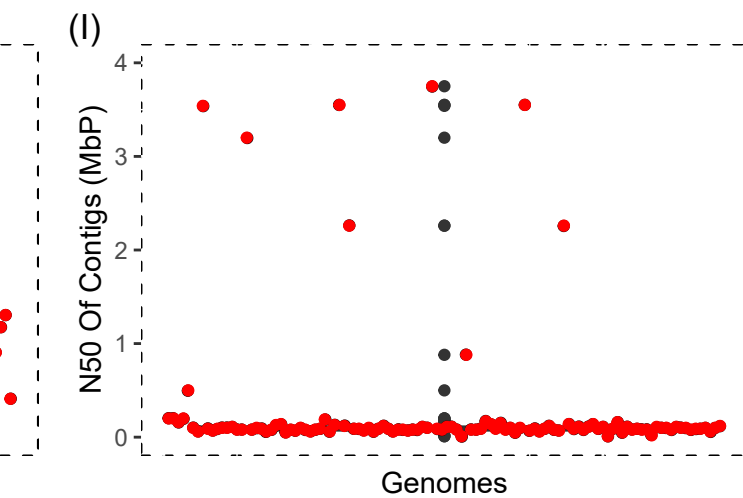

Supplement: Supplementary material 1 [file mgen-9-1071-s001.pdf]
